# Stabilization and Tracking of a Quadrotor Using Modified Sigmoid Sliding Mode Control

**DOI:** 10.3390/s22103618

**Published:** 2022-05-10

**Authors:** Mingyuan Hu, Kyunghyun Lee, Hyeongki Ahn, Ahyeong Choi, Hyunchang Kim, Kwanho You

**Affiliations:** 1 Department of Smart Fab. Technology, Sungkyunkwan University, Suwon 16419, Korea; hmy160831@g.skku.edu; 2 Department of Electrical and Computer Engineering, Sungkyunkwan University, Suwon 16419, Korea; naman2001@skku.edu (K.L.); ahk5721@skku.edu (H.A.); overcld7@skku.edu (A.C.); gusckddmldkt@g.skku.edu (H.K.)

**Keywords:** quadrotor, sliding mode control, nonlinear sliding surface, double-loop, tracking control

## Abstract

A modified sigmoid sliding mode control (MS-SMC) approach is proposed for stabilizing and tracking a quadrotor system with a nonlinear sliding surface, where the dynamics model is underactuated, highly coupled, and nonlinear. The constructed nonlinear sliding surface is based on the traditional sliding mode surface with a modified sigmoid function, allowing the initial value to quickly reach equilibrium. A new type of nonlinear SMC is applied for performance improvement of the quadrotor using the proposed modified sigmoid sliding surface. To control the quadrotor effectively, a double-loop control method is used to design the control rate, in which the position subsystem is the outer loop, and the attitude subsystem is the inner loop.With the Lyapunov function, the stability of the overall closed-loop system is ensured by stabilizing each subsystem step by step. Moreover, from a practical point of view, the system performance under the model uncertainties and external disturbances are also considered. The simulation results show that the proposed MS-SMC performs better than the conventional sliding mode control (CSMC) and the back-stepping sliding mode control (BS-SMC) in terms of stabilization and tracking against external disturbances.

## 1. Introduction

Unnamed aerial vehicles (UAVs) have advantages such as simple structure and high safety, and is very useful in disaster situations and agricultural fields. However, the performance of the PID controller is not satisfactory, to operate under uncertain environments. Therefore, in this paper, sliding mode control (SMC) was introduced to overcome the limitation of PID. SMC is an excellent robust controller and has advantages such as fast response speed and stability. However, in case of the widely used linear sliding mode control, the state cannot properly track the desired target. With the recent rapid expansion of the UAV market, quadrotor—a type of UAV—has attracted the attention of many researchers owing to its excellent control performance and convenience of vertical takeoff and landing. In contrast to fixed-wing aircraft, quadrotors have the advantages of vertical takeoff, landing, and fixed-point hovering. Compared to single-rotor helicopters, quadrotors have the advantages of a simple mechanical structure, high safety, and relatively low complexity of driving software owing to the absence of a tail rotor. Therefore, quadrotors have been widely used in logistics transportation [1], fire protection [2], and precision agriculture [3]. Among UAVs, the quadrotor is the most widely used rotor vehicle. There are various applications such as medical transportation, cargo delivery, emergency rescue, or resources delivery to isolated areas. The adaptability for industrial use proves its high prospects [4,5]. Since a quadrotor is a multi-input multi-output, highly coupled, under-driven system, stabilization and tracking control are the key issues associated with it. Although PID is a widely used controller, its linearity puts restrictions to control quadrotor systems with multiple inputs and multiple outputs properly. Therefore, the control output is limited, and it is difficult to fully derive the performance of the quadrotor. To overcome this problem, several controllers have been introduced. There are linear quadratic regulators, sliding mode control, back-stepping control, and adaptive control algorithms [6,7]. Among these controllers, SMC is a nonlinear controller that is robust against disturbance and shows excellent tracking ability.

As the advantage of conventional sliding mode control (CSMC) is that it can overcome the uncertainty of the system, it has robustness to disturbances and un-modeled dynamics [8,9,10]. In particular, it has a good control effect on nonlinear systems. Moreover, with the simple sliding mode structure algorithm, rapid response speed, robustness to external noise interference, and parameter perturbation, CSMC has been widely used in the field of quadrotor control. However, it is difficult for initial state to slide strictly along the sliding mode surface as it zigzags to approach the equilibrium point for a linear surface. The insecure sliding property cannot allow states to reach equilibrium within a finite time [11]. Therefore, Xiong created a terminal SMC [12]. However, the terminal sliding mode itself has a shortcoming of the singular problem [13]; hence, a nonsingular terminal sliding mode has been proposed to solve this problem in [14,15,16]. In [17], a fast terminal sliding mode control method for rigid manipulators was developed to achieve high accuracy tracking control. Based on this, a full-order end-sliding-mode control strategy was proposed to solve the chattering problem in [18]. Based on hybrid sliding mode algorithms, a fully robust back-stepping sliding mode controller has been constructed for both position control and attitude control [19,20]. In [21,22], an adaptive controller has been presented for attitude and position tracking based on combined integral sliding mode control, and the radial basic function neural network method, respectively. The unknown parameters are estimated through online using the neural network algorithm, requiring intensive computation. Hence, there exists a limit to be used for compact UAVs. For low-cost devices in measurement and control, a research suggested an accurate and economical method of estimating information on drone sensors [23]. As suggested in [23], if an advanced controller replaces the classical PID controller, a quadrotor with outstanding performance can be presented. In this study, we designed an SMC that enables quickly tracking and stabilizing the quadcopter. In general, quadrotor motion dynamics can be derived via the Newton–Euler and Euler–Lagrange methods. The Newton–Euler formalism provides physical insights through derivation. The Euler–Lagrange formalism provides the linkage between the classical framework and the Lagrangian or Hamiltonian method [24]. Herein, the dynamics of the quadrotor were formulated based on the Euler–Lagrange approach. The control input can be obtained through the SMC. The desired angle ($\psi_{d},\theta_{d}$) can be derived from the target position and control input. To provide rapid stability and trajectory tracking, a nonlinear sliding mode plane was designed to reduce the time to reach equilibrium. Moreover, dual-loop control [21,25,26] is used to design the control law, in which the attitude subsystem is the inner loop, and the position subsystem is the outer loop. The intermediate command signals $\psi_{d}$ and $\theta_{d}$ generated by the outer loop must be transmitted to the inner loop subsystem. The inner loop subsystem tracks two transmission signals through SMC. In dual-loop control, the attitude-angle tracking error of the inner loop system affects the stability of the outer loop, which induces the stability of the entire closed-loop control system. The stability problem is related to the inner and outer loop control, as the dynamic performance of the inner loop and the attitude-angle tracking error affect the stability of the outer loop, especially for an initial error, and, consequently, affect the stability of the entire closed-loop [27,28]. In this paper, position information is assumed to be detected by a three-axis accelerometer and is compared with location information of a virtual GPS. Attitude information is assumed to be detected through a three-axis gyroscope and compared with the desired angle values ($\theta_{d},\psi_{d}$) generated by the position controller. To improve the flying performance of the quadrotor, various sensors are mounted on the structure. Recently, a vision sensor was used to provide accurate location information. In addition, the altitude of quadrotor is precisely detected through a rangefinder to improve landing performance. Accurate attitude information is obtained through an additional inertia measurement unit along with gyroscope. Continuous direction update of quadrotor can also improve flight performance by using a magnetometer [29,30]. The simulation compared the performance of CSMC, BS-SMC, and MS-SMC presented in this paper. Position control and attitude subsystem control were performed under various conditions. Disturbance was applied to determine the robustness of the controller. Compared to CSMC and BS-SMC, MS-SMC reached the desired target with more stable performance and confirmed the lessened sensitivity to disturbance. In addition, the reaching speed of MS-SMC being faster than CMSC and BS-SMC shows that the modified sigmoid function designed in this paper functions properly.

In this study, we focus on designing a nonlinear SMC denoted by MS-SMC for position and altitude control with external disturbance on the dynamics in 6 DOF (x,y,z, roll, pitch, and yaw). The proposed nonlinear SMC is based on a modified sigmoid sliding surface. All controllers of this study are obtained from Lyapunov stability to ensure a fast stabilization and tracking in the presence of disturbances. We demonstrate the technological advantages of our approach through a comparison of simulation results from CSMC and BS-SMC. As a result, the chattering effect can be eliminated, resulting in a more stable performance. Moreover, the stabilization and tracking performance of a quadrotor are more robust and faster to adapt when the disturbance is bounded.

To design the nonlinear SMC based on a modified sigmoid sliding surface, this paper is organized as follows. In Section 2, the dynamical model of a quadrotor is presented. The control design method with a nonlinear sliding surface is described in Section 3 in addition to the control strategy for constructing the sliding mode controller of the position and attitude of a quadrotor. In Section 4, we demonstrate the effectiveness of modified sigmoid sliding mode control (MS-SMC) through simulation results. Finally, our conclusions are presented in Section 5.

## 2. Quadrotor Dynamic Modeling

**Figure 1:**
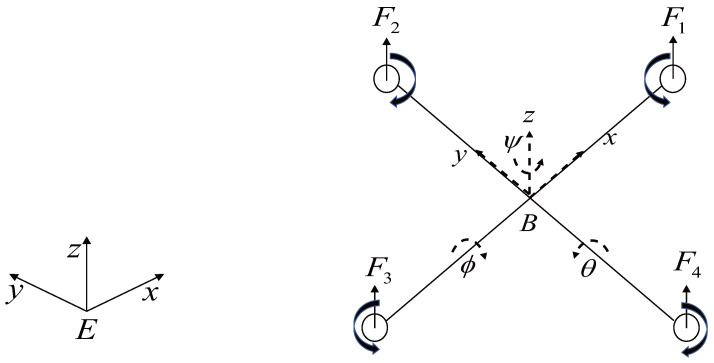
Coordinates of a quadrotor UAV.

The quadrotor used in this study was set up in the body frame and the earth frame as shown in Figure 1. More details of the configuration can be found in [31,32,33]. The dynamic model of the quadrotor can be derived via the Euler–Lagrange method, and a simplified model is found as follows:

(1) $$\begin{array}{cc} \ddot{x} & =u_{1}\left(\cos\phi \sin\theta \cos\psi +\sin\phi \sin\psi \right)-\frac{K_{1}\dot{x}}{m}+d_{x},\\ \ddot{y} & =u_{1}\left(\sin\phi \sin\theta \cos\psi -\cos\phi \sin\psi \right)-\frac{K_{2}\dot{y}}{m}+d_{y},\\ \ddot{z} & =u_{1}\cos\phi \cos\psi -g-\frac{K_{3}\dot{z}}{m}+d_{z},\\ \ddot{\theta } & =u_{2}-\frac{lK_{4}\dot{\theta }}{I_{1}}+d_{\theta },\\ \ddot{\psi } & =u_{3}-\frac{lK_{5}\dot{\psi }}{I_{2}}+d_{\psi },\\ \ddot{\phi } & =u_{4}-\frac{lK_{6}\dot{\phi }}{I_{3}}+d_{\phi }, \end{array}$$

where vector ${\left[x,y,z\right]}^{T}$ denotes the quadrotor position; vector ${\left[\phi ,\theta ,\psi \right]}^{T}$ represents the angles of roll, pitch, and yaw, respectively; $K_{i}$ denotes the drag coefficients; $I_{i}$ is the body inertia, *l* is the lever length; *g* is gravity, and *m* is the mass of the quadrotor. Here, $u_{i}\in R,i=1,2,3,4$, are the control inputs for a quadrotor, described as

(2) $$\begin{array}{c} \left[\begin{array}{c} u_{1}\\ u_{2}\\ u_{3}\\ u_{4} \end{array}\right]=\left[\begin{array}{c} \frac{\left(F_{1}+F_{2}+F_{3}+F_{4}\right)}{m}\\ \frac{l(-F_{1}-F_{2}+F_{3}+F_{4})}{I_{1}}\\ \frac{l(-F_{1}+F_{2}+F_{3}-F_{4})}{I_{2}}\\ \frac{C(F_{1}-F_{2}+F_{3}-F_{4})}{I_{3}} \end{array}\right], \end{array}$$

where $F_{i}$ is the thrust generated by four rotors, and *C* denotes the force-to-moment scaling factor. To consider unknown perturbations such as noise, uncertainty, unmodeled dynamics, and external disturbances in the real environment, the dynamic model of Equation (1) includes unknown perturbations ($d_{x},d_{y},d_{z},d_{\phi },d_{\theta },d_{\psi }$ ); thus, it leads to errors between the measured and estimated values of the states. Regarding the unknown disturbances, we redefine $d_{x},d_{y},d_{z},d_{\phi },d_{\theta }$, and $d_{\psi }$ after changing Equation (1) to the form of the state space as in Equation (5). To drive the control law, Equation (1) can be written in the state-space form of $\dot{x}=f\left(x,u\right)$. Let $x\in {\left[x_{1},x_{2},\cdot \cdot \cdot ,x_{12}\right]}^{T}$ be a state variable vector, defined as:

(3) $$\begin{array}{cc} x_{1} & =x,x_{2}=\dot{x},x_{3}=y,x_{4}=\dot{y},x_{5}=z,x_{6}=\dot{z},\\ x_{7} & =\theta ,x_{8}=\dot{\theta },x_{9}=\psi ,x_{10}=\dot{\psi },x_{11}=\phi ,x_{12}=\dot{\phi }. \end{array}$$

In Equation (1), the mechanical structure can be written as

(4) $$\begin{array}{cc} a_{1} & =\frac{K_{1}}{m},a_{2}=\frac{K_{2}}{m},a_{3}=\frac{K_{3}}{m},\\ a_{4} & =\frac{lK_{4}}{I_{1}},a_{5}=\frac{lK_{5}}{I_{2}},a_{6}=\frac{lK_{6}}{I_{3}},\\ u_{x} & =\cos x_{11}\sin x_{7}\cos x_{9}+\sin x_{11}\sin x_{9},\\ u_{y} & =\sin x_{11}\sin x_{7}\cos x_{9}-\cos x_{11}\sin x_{9},\\ u_{z} & =\cos x_{{}_{11}}\cos x_{9}. \end{array}$$

In Equation (4), $a_{1}$ through $a_{6}$, are parameters comprising body inertia, lever length, and rotor inertia. $u_{x}$ and $u_{y}$ are the control inputs of the quadrotor, using roll, pitch, and yaw. From Equations (1) and (3), the dynamics of the quadrotor can be obtained using Equation (5):

(5) $$\begin{array}{c} \dot{x}=f\left(x,u\right)=\left[\begin{array}{c} x_{2}\\ u_{1}\left(u_{x}+\Delta u_{1}\right)-\left(a_{1}+\Delta a_{1}\right)x_{2}+d_{1}+n_{1}\\ x_{4}\\ u_{1}\left(u_{y}+\Delta u_{2}\right)-\left(a_{2}+\Delta a_{2}\right)x_{4}+d_{2}+n_{2}\\ x_{6}\\ u_{1}\left(u_{z}+\Delta u_{3}\right)-g-\left(a_{3}+\Delta a_{3}\right)x_{6}+d_{3}+n_{3}\\ x_{8}\\ u_{2}\left(1+\Delta u_{4}\right)-\left(a_{4}+\Delta a_{4}\right)x_{8}+d_{4}+n_{4}\\ x_{10}\\ u_{3}\left(1+\Delta u_{5}\right)-\left(a_{5}+\Delta a_{5}\right)x_{10}+d_{5}+n_{5}\\ x_{12}\\ u_{4}\left(1+\Delta u_{6}\right)-\left(a_{6}+\Delta a_{6}\right)x_{12}+d_{6}+n_{6} \end{array}\right] \end{array}$$

From a practical point of view, we updated Equation (1) to state-space form and added environmental noise, uncertainty, unmodeled dynamic, and external disturbances in Equation (5). $\Delta u_{i},\Delta a_{i},$ and $d_{i}$, i=1,2,⋯,6 are the nonlinear functions that introduce the system uncertainties, unmodeled dynamic, and external disturbance, respectively. $\;n_{i},\;\;i=1,2,\cdots ,6$ is a term that simulates a noisy environment. To handle better the system uncertainties, unmodeled dynamic, external disturbances, and environmental noise are redefined as $d_{x}=u_{1}\Delta b_{1}-\Delta a_{1}x_{2}+d_{1}+n_{1}$, $d_{y}=u_{1}\Delta b_{2}-\Delta a_{2}x_{4}+d_{2}+n_{2}$, $d_{z}=u_{1}\Delta b_{3}-\Delta a_{3}x_{6}+d_{3}+$$n_{3}$, $d_{\theta }=u_{2}\Delta b_{4}-\Delta a_{4}x_{8}+d_{4}+n_{4}$, $d_{\psi }=u_{3}\Delta b_{5}-\Delta a_{5}x_{10}+d_{5}$$+n_{5}$, $d_{\phi }=u_{3}\Delta b_{5}-\Delta a_{6}x_{12}+$$d_{5}+n_{6}$. Therefore, Equation (5) can be considered as:

(6) $$\begin{array}{c} \dot{x}=f\left(x,u\right)=\left[\begin{array}{c} x_{2}\\ u_{1}u_{x}-a_{1}x_{2}+d_{x}\\ x_{4}\\ u_{1}u_{y}-a_{2}x_{4}+d_{y}\\ x_{6}\\ u_{1}u_{z}-g-a_{3}x_{6}+d_{z}\\ x_{8}\\ u_{2}-a_{4}x_{8}+d_{\theta }\\ x_{10}\\ u_{3}-a_{5}x_{10}+d_{\psi }\\ x_{12}\\ u_{4}-a_{6}x_{12}+d_{\phi } \end{array}\right]. \end{array}$$

**Figure 2:**
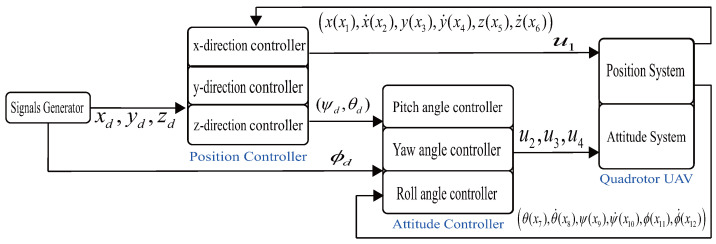
Control scheme of quadrotor.

The structure of the entire control system is shown in Figure 2. The control system in Equation (6) belongs to the inner and outer loop control system and adopts the double-loop control method. The position subsystem is the outer loop, and the attitude subsystem comprises the inner loop. The outer loop generates two intermediate command signals $\left(\psi_{d},\theta_{d}\right)$ and transmits the results to the inner loop. The inner loop tracks these two intermediate commands using the SMC law.

## 3. Nonlinear Sliding Mode Control Design

### 3.1. Nonlinear Sliding Surface

**Figure 3:**
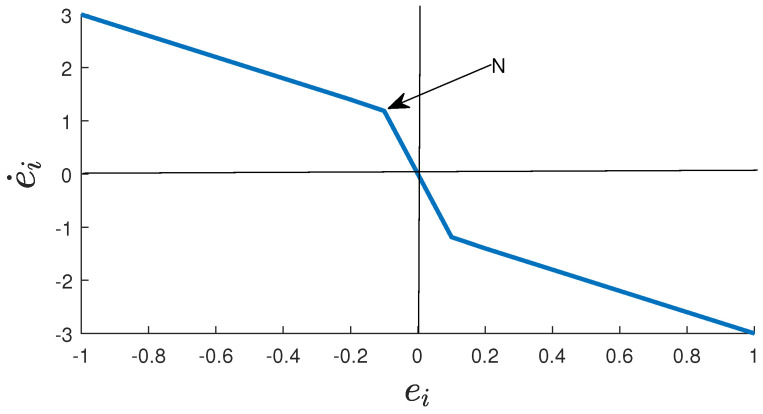
The proposed modified sigmoid sliding surface.

As a CSMC, a linear sliding surface was applied owing to the simple design procedure. For better performance, we consider the nonlinear sliding surface of a quadrotor controller for fast transient response. In Equations (7) and (8), $\sigma \left(e_{i},t\right)$ indicates the sliding plane. The sliding surface function allows the state to move swiftly to the desired state in SMC. The traditional sliding plane can be written in the following form:

(7) $$\begin{array}{cc} \sigma (e_{i},t) & =\lambda_{i}e_{i}+{\dot{e}}_{i},\\ \;\;\;\;\;\;\;\;\;\;\;\;\;\;\;\;\;\;\; & =\lambda_{i}\left(x_{i}-x_{id}\right)+{\dot{x}}_{i}-{\dot{x}}_{id}, \end{array}$$

where $\lambda_{i}$ is a positive integer, and $e_{i}$ denotes the error between the state value ($x_{i}$) and the desired value ($x_{id}$). For a fast stabilization and tracking response, a new nonlinear sliding surface is proposed as follows:

(8) $$\begin{array}{c} \sigma \left(e_{i},t\right)=-b_{i}\frac{1-\varepsilon^{-c_{i}e_{i}}}{1+\varepsilon^{-c_{i}e_{i}}}+h_{i}e_{i}+{\dot{e}}_{i}, \end{array}$$

where ε is an exponential function, and $b_{i},c_{i}$, and $h_{i}$ are positive constants. The sigmoid function has the same basic form as $e^{x}/\left(e^{x}+1\right)$. In this paper, it is modified as in Equation (8) to increase the reaching speed to equilibrium. Figure 3 shows the modified sigmoid sliding surface. In Figure 3, parameters $b_{i}$, $c_{i}$, and $h_{i}$ are set to 1, 50, and 2, respectively. The sliding mode plane can be divided into two parts. The first part is from infinity to *N*, and the second part is from *N* to 0. The first part is the linear plane that is controlled by $h_{i}e_{i}$ from Equation (8), and the second part is the nonlinear part that is controlled by $-b_{i}\left(1-\varepsilon^{-c_{i}e_{i}}\right)/\left(1+\varepsilon^{-c_{i}e_{i}}\right)$ in Equation (8). When the initial value reaches the first part of the sliding mode plane, it will first move to 0 along the linear sliding mode plane, and then accelerate to 0 along the nonlinear sliding mode plane after reaching point *N*. When the initial value reaches the second part of the sliding mode plane, it will converge to 0 directly along the nonlinear sliding mode plane. It can be seen from Figure 3 that the inclination angle of the second part is much larger than that of the first part, so the convergence speed is much faster than the speed of the first part. Contrary to the traditional linear sliding mode plane, the sliding mode plane that we designed has nonlinear acceleration parts. Therefore, MS-SMC converges faster than the CSMC. We design the controller according to the sliding surface in Equation (8).

### 3.2. Position Subsystem Control

Owing to the complex characteristics of the quadrotor system, the proposed control scheme adopts a feedback dual-loop system, as shown in Figure 2. The position system is in the outer loop, and the attitude subsystem is in the inner loop. First, the position system controller was designed. From Equation (1), the subsystems $u_{x},u_{y},u_{z}$ for position control can be defined as

(9) $$\begin{array}{cc} u_{x} & =u_{1}\left(\cos\phi \sin\theta \cos\psi +\sin\phi \sin\psi \right),\\ u_{y} & =u_{1}\left(\sin\phi \sin\theta \cos\psi -\cos\phi \sin\psi \right),\\ u_{z} & =u_{1}\cos\phi \cos\psi , \end{array}$$

where $u_{1}$ is associated with pitch and yaw angle. Using Equation (9), the formula for Equation (1) can be modified. The position-related parts in Equation (1) can be represented in the following form:

(10) $$\begin{array}{cc} \ddot{x} & =u_{x}-a_{1}\dot{x}+d_{x},\\ \ddot{y} & =u_{y}-a_{2}\dot{y}+d_{y},\\ \ddot{z} & =u_{z}-g-a_{3}\dot{z}+d_{z}. \end{array}$$

To overcome the time delay owing to the deceleration curve in SMC, the nonlinear sliding surface that reduces the convergence time with a modified deceleration curve was proposed [34]. With Equation (8), the novel modified sigmoid sliding surfaces can achieve the fast-tracking trajectory owing to a nonlinear term in the sliding surfaces. Using the new nonlinear sliding surface in Equation (8) to design a sliding mode controller, the nonlinear sliding surfaces can be represented as:

(11) $$\begin{array}{cc} e_{1} & =x_{1}-x_{1d},\;\;{\dot{e}}_{1}=x_{2}-x_{2d},\\ e_{2} & =x_{3}-x_{3d},\;\;{\dot{e}}_{2}=x_{4}-x_{4d},\\ e_{3} & =x_{5}-x_{5d},\;\;{\dot{e}}_{3}=x_{6}-x_{6d,} \end{array}$$

where $e_{1}$, $e_{2}$, and $e_{3}$ are the errors of $x_{1}$, $x_{3}$, and $x_{5}$, respectively; ${\dot{e}}_{1}$, ${\dot{e}}_{2},$ and ${\dot{e}}_{3}$ are the errors of $x_{2}$, $x_{4}$, and $x_{6}$, respectively:

(12) $$\begin{array}{cc} \sigma_{x} & =-b_{1}\frac{1-\varepsilon^{-c_{1}e_{1}}}{1+\varepsilon^{-c_{1}e_{1}}}+h_{1}e_{1}+{\dot{e}}_{1},\\ \sigma_{y} & =-b_{2}\frac{1-\varepsilon^{-c_{2}e_{2}}}{1+\varepsilon^{-c_{2}e_{2}}}+h_{2}e_{2}+{\dot{e}}_{2},\\ \sigma_{z} & =-b_{3}\frac{1-\varepsilon^{-c_{3}e_{3}}}{1+\varepsilon^{-c_{3}e_{3}}}+h_{3}e_{3}+{\dot{e}}_{3}, \end{array}$$

where $\sigma {}_{x},\sigma {}_{y}$, and $\sigma_{z}$ are the three sliding surfaces for $u_{x},u_{y}$, and $u_{z}$, respectively. For the first position subsystem, the controller can be designed as follows:

(13) $$\begin{array}{c} u_{x}=-\left(b_{1}\frac{\varepsilon^{-c_{1}e_{1}}}{{\left(1+\varepsilon^{-c_{1}e_{1}}\right)}^{2}}c_{1}{\dot{e}}_{1}+h_{1}{\dot{e}}_{1}-a_{1}x_{2}-{\dot{x}}_{2d}+\eta_{1}\mathrm{sgn}\left(\sigma_{x}\right)+\alpha_{1}\sigma_{x}\right), \end{array}$$

where $\eta_{1}$ and $\alpha_{1}$ have conditions of $\eta_{1}\geq d_{x}$ and, $\alpha_{1}>0$, respectively.

**Proposition** **1.** *For the position system in x-direction described by Equation (10), if the sliding mode surface $\sigma (e_{i},t)$ is selected as in Equation (8), the quadrotor in x-direction guarantees stability and tracks the desired value fast.*

**Proof.**  The stability of $u_{x}$ can be proved by the Lyapunov function of $V\left(\sigma_{x}\right)=\sigma_{x}^{2}/2$ as

(14) $$\begin{array}{cc} \dot{V}\left(\sigma_{x}\right) & =\sigma_{x}{\dot{\sigma }}_{x}\\ \;\;\;\;\;\;\;\;\;\;\;\;\;\;\;\;\;\; & =\sigma_{x}\left(b_{1}\frac{\varepsilon^{-c_{1}e_{1}}}{{\left(1+\varepsilon^{-c_{1}e_{1}}\right)}^{2}}c_{1}{\dot{e}}_{1}+h_{1}{\dot{e}}_{1}+u_{x}-a_{1}x_{2}+d_{x}-{\dot{x}}_{2d}\right). \end{array}$$

Substituting Equation (12) into Equations (13) and (14) can be simplified as follows:

(15) $$\begin{array}{cc} \dot{V}\left(\sigma_{x}\right) & =\sigma_{x}\left[-\eta_{1}\mathrm{sgn}\left(\sigma_{x}\right)-\alpha_{1}\sigma_{x}\right]\\ \;\;\;\;\;\;\;\;\;\;\;\;\;\;\;\;\;\;\; & =-\eta_{1}\left|\sigma_{x}\right|-\alpha_{1}\sigma_{x}^{2}<0. \end{array}$$

This completes the proof of stability. By following the same design procedures for $u_{y}$ and $u_{z}$, we obtain:

(16) $$\begin{array}{cc} u_{y} & =-\left(b_{2}\frac{\varepsilon^{-c_{2}e_{2}}}{{\left(1+\varepsilon^{-c_{2}e_{2}}\right)}^{2}}c_{2}{\dot{e}}_{2}+h_{2}{\dot{e}}_{2}-a_{2}x_{4}-{\dot{x}}_{4d}+\eta_{2}\mathrm{sgn}\left(\sigma_{y}\right)+\alpha_{2}\sigma_{y}\right), \end{array}$$

(17) $$\begin{array}{cc} u_{z} & =-\left(b_{3}\frac{\varepsilon^{-c_{3}e_{3}}}{{\left(1+\varepsilon^{-c_{3}e_{3}}\right)}^{2}}c_{3}{\dot{e}}_{3}+h_{3}{\dot{e}}_{3}-a_{3}x_{6}-g-{\dot{x}}_{6d}+\eta_{3}\mathrm{sgn}\left(\sigma_{z}\right)+\alpha_{3}\sigma_{z}\right), \end{array}$$

where $\eta_{2}$ and $\alpha_{2}$ have conditions that are $\eta_{2}\geq d_{y}$ and $\alpha_{2}>0$, respectively; $\eta_{3}$ and $\alpha_{3}$ have conditions that are $\eta_{3}\geq d_{z}$ and $\alpha_{3}>0$, respectively. □

### 3.3. Virtual Attitude Angles

To achieve θ and ψ tracking for $\theta_{d}$ and $\psi_{d}$, we need to obtain the solution in a closed analytic form. The subsystem łfor and in Equation (9) can be expressed in matrix form:

(18) $$\begin{array}{c} \left[\begin{array}{c} u_{x}\\ u_{y} \end{array}\right]=\left[\begin{array}{cc} \cos\phi_{d} & \sin\phi_{d}\\ \sin\phi_{d} & -\cos\phi_{d} \end{array}\right]\left[\begin{array}{c} \sin\theta_{d}\cos\psi_{d}\\ \;\;\;\;\;\;\;\sin\psi_{d} \end{array}\right]u_{1}. \end{array}$$

For the first row of Equation (18), $\psi_{d}$ can be found as:

(19) $$\psi_{d}=\arctan\left(\frac{\sin\phi_{d}\cos\phi_{d}u_{x}-\cos^{2}\phi_{d}u_{y}}{u_{z}}\right).$$

For the second row of Equation (18), $\theta_{d}$ can be found as:

(20) $$sin\theta_{d}=\frac{\cos\phi_{d}\left(\cos\phi_{d}u_{x}+\sin\phi_{d}u_{y}\right)}{u_{z}}.$$

Since the value of the sine function is between −1 and 1, the value of $\sin\theta_{d}$ is bounded as $-1<\sin\theta_{d}<1.$ To prevent $sin\theta_{d}$ from taking a real value out of the [−1, +1] range, we define an intermediate value *H*, which is $\cos\phi_{d}\left(\cos\phi_{d}u_{x}+\sin\phi_{d}u_{y}\right)/u_{z}$. When *H* is out of the range [−1, +1], $\theta_{d}$ does not exist. We thus assign values to achieve the continuity of Equation (20), i.e., if H<−1, $\sin\theta_{d}=-1$ and $\theta_{d}=-\pi /2$; if H>1, $\sin\theta_{d}=1$ and $\theta_{d}=\pi /2$; finally, when |H|≤1, $\theta_{d}$ is calculated as follows:

(21) $$\theta_{d}=\arcsin\left[\frac{\cos\phi_{d}\left(\cos\phi_{d}u_{x}+\sin\phi_{d}u_{y}\right)}{u_{z}}\right],$$

Since $u_{1}$ is related to roll and yaw angle, after obtaining $\theta_{d}$ and $\psi_{d}$, the control law of position $u_{1}$ is as follows:

(22) $$u_{1}=\frac{u_{z}}{\cos\phi_{d}\cos\psi_{d}}.$$

### 3.4. Attitude Subsystem Control

As the outer loop system of the two angles (roll and yaw) generated by the position system needs to be processed in the inner loop, three inputs (roll, pitch, and yaw) are passed to the position system. The design of the attitude subsystem is important for deciding a control input that is closely related to the location output. By using the new nonlinear sliding surface in Equation (8) to design the attitude controller, the nonlinear sliding surface can be represented as:

(23) $$\begin{array}{cc} e_{4} & =x_{7}-x_{7d},\;\;{\dot{e}}_{4}=x_{8}-x_{8d},\\ e_{5}\;\; & =x_{9}-x_{9d},\;\;{\dot{e}}_{5}\;\;\;=x_{10}-x_{10d},\\ e_{6}\;\; & =x_{11}-x_{11d},\;\;{\dot{e}}_{6}=x_{12}-x_{12d,} \end{array}$$

where $e_{4},e_{5}$, and $e_{6}$ are the errors of $x_{7},x_{9}$, and $x_{11}$, respectively, ${\dot{e}}_{4},{\dot{e}}_{5}$, and ${\dot{e}}_{6}$ are the errors of $x_{8},x_{10}$, and $x_{12}$, respectively.

(24) $$\begin{array}{cc} \sigma_{\theta } & =-b_{4}\frac{1-\varepsilon^{-c_{4}e_{4}}}{1+\varepsilon^{-c_{4}e_{4}}}+h_{4}e_{4}+{\dot{e}}_{4},\\ \sigma_{\psi } & =-b_{5}\frac{1-\varepsilon^{-c_{5}e_{5}}}{1+\varepsilon^{-c_{5}e_{5}}}+h_{5}e_{5}+{\dot{e}}_{5},\\ \sigma_{\phi } & =-b_{6}\frac{1-\varepsilon^{-c_{6}e_{6}}}{1+\varepsilon^{-c_{6}e_{6}}}+h_{6}e_{6}+{\dot{e}}_{6}, \end{array}$$

$\sigma_{\theta },\sigma_{\psi }$, and $\sigma_{\phi }$ are three sliding surfaces for $u_{2},u_{3}$, and $u_{4}$, respectively. Following the same design procedures as the secondary position subsystem, an attitude controller can be designed. Therefore, the controllers for the attitude angle (θ, ψ, ϕ) are as follows:

(25) $$\begin{array}{cc} u_{2} & =-\left(b_{4}\frac{\varepsilon^{-c_{4}e_{4}}}{{\left(1+\varepsilon^{-c_{4}e_{4}}\right)}^{2}}c_{4}{\dot{e}}_{4}+h_{4}{\dot{e}}_{4}-a_{4}x_{8}-{\dot{x}}_{8d}+\eta_{4}\mathrm{sgn}\left(\sigma_{\theta }\right)+\alpha_{4}\sigma_{\theta }\right), \end{array}$$

(26) $$\begin{array}{cc} u_{3} & =-\left(b_{5}\frac{\varepsilon^{-c_{5}e_{5}}}{{\left(1+\varepsilon^{-c_{5}e_{5}}\right)}^{2}}c_{5}{\dot{e}}_{5}+h_{5}{\dot{e}}_{5}-a_{5}x_{10}-{\dot{x}}_{10d}+\eta_{5}\mathrm{sgn}\left(\sigma_{\psi }\right)+\alpha_{5}\sigma_{\psi }\right), \end{array}$$

(27) $$\begin{array}{cc} u_{4} & =-\left(b_{6}\frac{\varepsilon^{-c_{6}e_{6}}}{{\left(1+\varepsilon^{-c_{6}e_{6}}\right)}^{2}}c_{6}{\dot{e}}_{6}+h_{6}{\dot{e}}_{6}-a_{6}x_{12}-{\dot{x}}_{12d}+\eta_{6}\mathrm{sgn}\left(\sigma_{\phi }\right)+\alpha_{6}\sigma_{\phi }\right), \end{array}$$

where $\eta_{4},\eta_{5}$, and $\eta_{6}$ have conditions that $\eta_{4}\geq d_{\theta }$, $\eta_{5}\geq d_{{}^{\psi }},\eta_{6}\geq d_{\phi }$, and $\alpha_{4},\alpha_{5}$, and $\alpha_{6}$ are positive. $u_{3}$ is the controller related to the attitude angle of pitch, and $u_{4}$ controls the attitude angle of yaw, which can realize the fast convergence of θ and ψ. Moreover, $u_{2}$ controls the roll angle ϕ, which can track the desired angle of $\phi_{d}$. Due to the existence of the discontinuous switching function sgn(.) in Equations (13), (16), (17) and (25)–(27), the chattering problem occurs after the initial value reaches the sliding surface; therefore, the nonlinear quadrotor system with uncertainty may appear to be an undesired response. To solve this problem, the function sgn(.) can be replaced by the following continuous saturation function:

(28) $$\begin{array}{cc} sat(\sigma ) & =\left\{\begin{array}{c} \mathrm{sgn}(\sigma ),\;\;\;\;\;\;\;|\sigma |>\Lambda ,\\ \frac{\sigma }{\Lambda },\;\;\;\;\;\;\;\;\;\;\;\;\;\;\;\;\;\;\;\;\left|\sigma \right|\leq \Lambda ,\; \end{array}\right. \end{array}$$

where Λ is the boundary layer thickness.

The Lyapunov candidate function for overall closed-loop system in Equation (5) is selected as

(29) $$V_{overall}=\frac{1}{2}\left(\sigma_{x}^{2}+\sigma_{y}^{2}+\sigma_{z}^{2}+\sigma_{\theta }^{2}+\sigma_{\psi }^{2}+\sigma_{\phi }^{2}\right).$$

The derivative of Equation (29) with respect to time can be written as Equation (30) with $u_{x},u_{y},u_{z},u_{\theta },u_{\psi },\;\mathrm{and}\;\;u_{\phi }$ being substituted by Equations (13), (16), (17) and (25)–(27), respectively. Therefore, the stability of overall closed-loop system is guaranteed by Equations (29) and (30), ensuring trajectory tracking capability of position and attitude subsystem:

(30) $${\dot{V}}_{overall}=\sigma_{x}{\dot{\sigma }}_{x}+\sigma_{y}{\dot{\sigma }}_{y}+\sigma_{z}{\dot{\sigma }}_{z}+\sigma_{\theta }{\dot{\sigma }}_{\theta }+\sigma_{\psi }{\dot{\sigma }}_{\psi }+\sigma_{\phi }{\dot{\sigma }}_{\phi }\leq 0.$$

## 4. Simulations

In this section, a simulation is performed to confirm the effectiveness of the quadrotor controller based on the proposed MS-SMC. MS-SMC exhibited high performance compared with CSMC and BS-SMC. To prove the feasibility of the proposed control algorithm, we used MATLAB/Simulink and chose ODE45 as a solverin Simulink. In our simulation, $d_{x},d_{y},d_{z},d_{\phi },d_{\theta }$, and $d_{\psi }$ are set to −0.2+0.4·rand(t). The parameters of the UAV model are listed in Table 1. $h_{1},h_{2}$, and $h_{3}$ are the parameters of the position controller, and the values are set to 1.1. $h_{4},h_{5}$, and $h_{6}$ are related to the parameters of the attitude controller, and the values are all 50. $\alpha_{1},\alpha_{2}$, and $\alpha_{3}$ are parameters related to the control rate of the position controller, with the value of 0.10. $\alpha_{4},\alpha_{5}$, and $\alpha_{6}$ are parameters related to the attitude control rate, with the value of 0.10. η is a parameter to adjust the chattering of the sliding mode controller. The position parameters are $\eta_{1},\eta_{2}$, and $\eta_{3}$, with the value of 0.1. The attitude controller’s parameters $\eta_{1},\eta_{2}$, and $\eta_{3}$ with the value of 50. Moreover, the boundary layer thickness is set to 0.2 in the saturation function. The angle that we track is 72°, and the desired altitude in the *z*-axis direction is 3.0 m.

**Figure 4:**
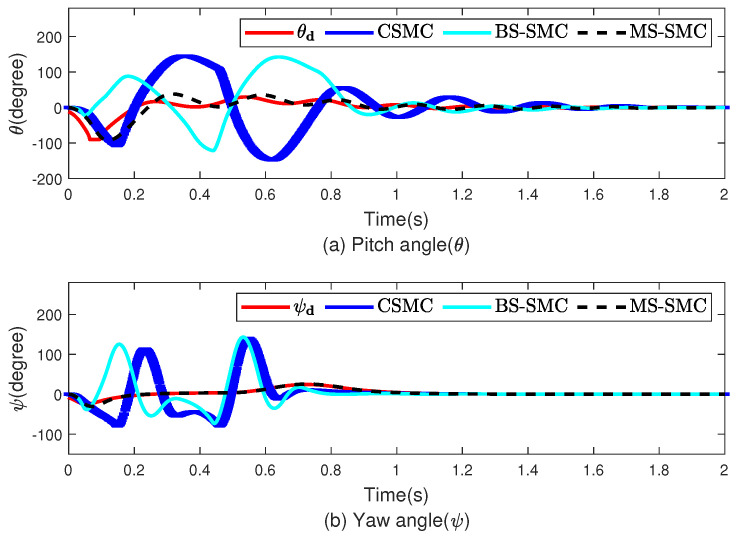
Stabilization of quadrotor using MS-SMC, CSMC, and BS-SMC.

**Figure 5:**
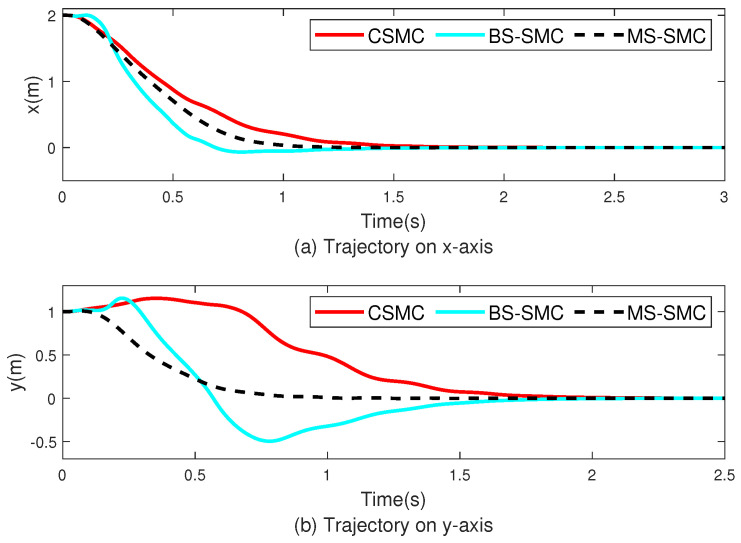

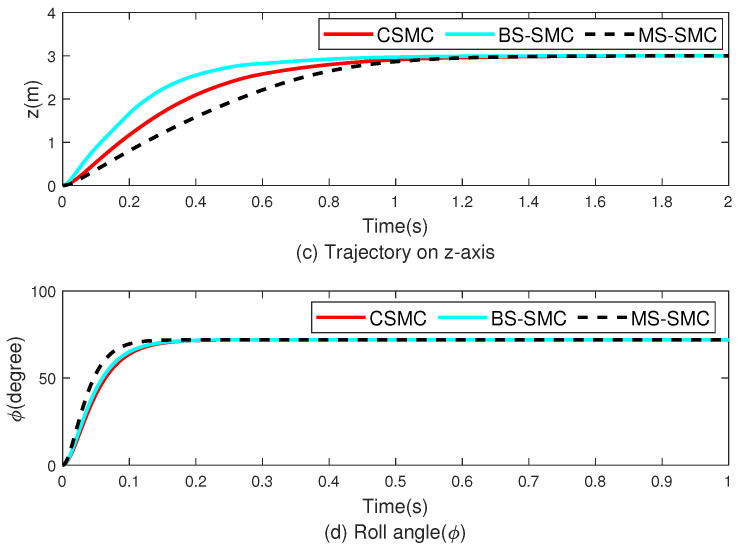
Tracking of quadrotor for x,y,z, and ϕ.

**Figure 6:**
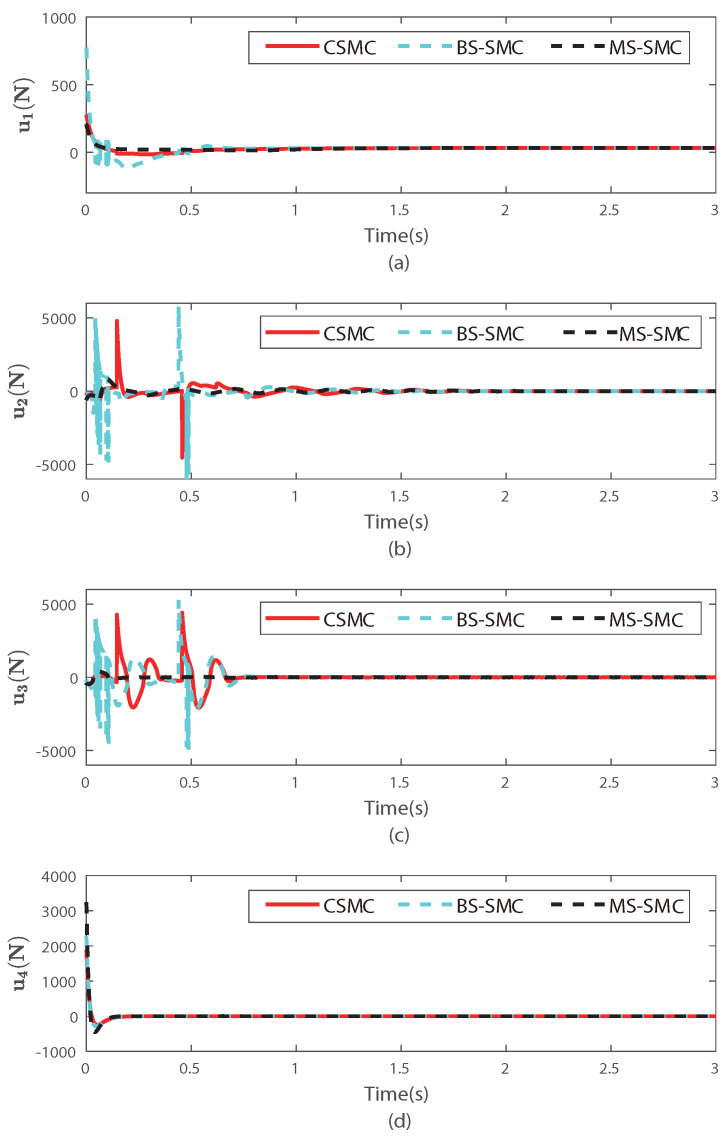
Control inputs of overall system: (**a**) control input for lifting force, (**b**) control input for pitch angle, (**c**) control input for roll angle, and (**d**) control input for yaw angle.

Figure 4 and Figure 5 show the performances on tracking and stability, respectively. Figure 4 shows the simulation results for stability, which correspond to the pitch and yaw angles. $\theta_{d}$ and $\psi_{d}$ are derived from x,y, and *z* in the position subsystem and are related to the roll value. Therefore, $\theta_{d}$ and $\psi_{d}$ change over time, as shown in Figure 4. Figure 4a,b show the tracking trajectories of attitude angles (θ,ψ) controlled by CSMC, BS-SMC, and MS-SMC, respectively. In Figure 4, the red, blue, cyan, and black dashed lines represent the desired angle, the quadrotor angles of CSMC, BS-SMC, and MS-SMC, respectively. As shown in Figure 4, the CSMC and BS-SMC have a large deviation, and the MS-SMC follows the desired angle of $\theta_{d}$ and $\psi_{d}$ with less fluctuation. In Figure 4a, the pitch angle θ of each method tracks its desired signal $\theta_{d}$ at *t* = 1.2 s by CSMC, at *t* = 1.1 s by BS-SMC, and at *t* = 0.8 s by MS-SMC. In Figure 4b, the yaw angle ψ of each method tracks its desired signal $\psi_{d}$ at *t* = 1 s by CSMC and BS-SMC, and at *t* = 0.1 s by MS-SMC. Therefore, tracking time is effectively lessened by MS-SMC. Figure 5 shows the tracking of degrees of freedom such as x,y,z, and roll angle, which are represented by subgraphs (a)–(d), respectively, and the designed MS-SMC is compared with CSMC and BS-SMC. Figure 5a,b describe the movement in the *x* and *y* directions, respectively.In Figure 5, the red, cyan, and black dashed lines represent CSMC, BS-SMC, and MS-SMC, respectively. Moreover, initial values are set to x(0)=2.0 m and y(0)=1.0 m. Both values were set to finally reach the position of 0 m. These changes can clearly show the control performance of each controller. The results show that MS-SMC reaches the equilibrium point faster than CSMC and BS-SMC. Figure 5c,d describe the trajectory in the *z*-axis and roll angle, respectively. In Figure 5a, the desired trajectory of *x* is tracked after 1.5 s by CSMC, and 1.4 s by BS-SMC. For MS-SMC, the result is much better with a tracking time of 1.2 s. The position of *y* reaches the target value of 0 m at *t* = 2.0 s by CSMC, at *t* = 1.8 s by BS-SMC, and at *t* = 0.8 s by MS-SMC in Figure 5b. In Figure 5c, CSMC, BS-SMC, and MS-SMC take the same time of 1.4 s to reach the desired value in the *z*-direction. In Figure 5d, the desired angle of ϕ is tracked by MS-SMC after 0.14 s while the tracking time is 0.2 s for CSMC and BS-SMC. We checked the tracking performance in the *z*-axis and roll angle. After setting the initial value in the *z*-axis to 0, the target value is put as 3.0 m. At this time, the controller has the role of adjusting the roll angle from the initial value $0^{\circ }$ to $72^{\circ }$. In this paper, it can be checked that MS-SMC reaches the target value quickly compared to CSMC. As disturbances are applied, the system controlled by the CSMC and BS-SMC becomes unstable. On the contrary, MS-SMC makes it easier to maintain the stability after reaching the target. The simulation results show that MS-SMC reaches the target value faster than CSMC and BS-SMC. Figure 6 shows the control input comparison for CMSC, BS-SMC, and MS-SMC. The control input $u_{1}$ represents the total force applied for the altitude control of the quadrotor. Other control inputs $\left(u_{1},u_{2},u_{3}\right)$ are the force values for control of pitch, yaw, and roll angles, respectively. MS-SMC demonstrates better control behavior in Figure 6.

## 5. Conclusions

In this study, the MS-SMC based on a new nonlinear sliding surface was applied to improve the stabilization and tracking of a quadrotor.In this study, we applied the MS-SMC based on a new nonlinear sliding surface to improve the stabilization and tracking of a quadrotor. Compared with CSMC and BS-SMC, a nonlinear sliding surface can reduce the time required to reach equilibrium. The position control $\left(u_{x},u_{y},u_{z},u_{1}\right)$ can be derived from the position subsystem using the proposed MS-SMC algorithm. Attitude control $\left(u_{2},u_{3},u_{4}\right)$ can be obtained by the attitude subsystem using the proposed MS-SMC. The desired angles of roll and pitch are obtained by the control inputs of *x*, *y*, and *z*. According to our design method, the pitch and yaw angles (θ,ψ) can converge quickly to $\theta_{d}$ and $\psi_{d}$. Similarly, x,y,z, and ϕ can achieve good tracking effects compared to the CSMC and BS-SMC.

## Figures and Tables

**Table 1:** Numerical parameters of the quadrotor model.

Parameter	Value	Unit
$I_{1}$,$I_{2}$	1.25	$\left[{\mathrm{Ns}}^{2}/\mathrm{rad}\right]$
$I_{3}$	2.5	$\left[{\mathrm{Ns}}^{2}/\mathrm{rad}\right]$
*m*	2	[kg]
$K_{1}=K_{2}=K_{3}$	0.010	[Ns/m]
$K_{4}=K_{5}=K_{6}$	0.012	[Ns/m]
*l*	0.2	[m]
*g*	9.8	$\left[m/s^{2}\right]$
x(0)	2	[m]
y(0)	1	[m]
z(0)	0	[m]
θ(0)=ϕ(0)=ψ(0)	0	[degree]

## Data Availability

Data sharing not applicable.
